# Host Usage in *Aedes aegypti* from Houston, Texas, and Phoenix, Arizona, Using Third-Generation Sequencing Blood Meal Analysis

**DOI:** 10.3390/insects17020175

**Published:** 2026-02-05

**Authors:** Brittani A. Ciomperlik, Edwin R. Burgess, Neil D. Sanscrainte, Mba-Tihssommah Mosore, John Townsend, James B. Will, Nicole Busser, Alden S. Estep

**Affiliations:** 1 Entomology and Nematology Department, University of Florida, Gainesville, FL 32611, USA; ciomperlikb@ufl.edu (B.A.C.); edwinburgess@ufl.edu (E.R.B.IV); mmosore@ufl.edu (M.-T.M.); 2 Mosquito & Fly Research Unit, Center for Medical, Agricultural, and Veterinary Entomology, Agricultural Research Service, United States Department of Agriculture, Gainesville, FL 32608, USA; neil.sanscrainte@usda.gov; 3 Vector Control Division, Maricopa County Environmental Services, Phoenix, AZ 85003, USA; john.townsend@maricopa.gov (J.T.); james.will@maricopa.gov (J.B.W.); nicole.busser@maricopa.gov (N.B.)

**Keywords:** *Aedes aegypti*, mosquito, blood meal analysis, third-generation sequencing, nanopore, host usage

## Abstract

The ecology and establishment of *Aedes aegypti* in tropical, sub-tropical, and temperate areas makes them one of the most medically relevant mosquito species. While they have been reported to be highly anthropophilic, several studies indicate a broader host range. They also reportedly take multiple blood meals between gonotrophic cycles, which makes determination of host usage difficult when using common blood meal analysis methods. In this study, we examined host usage of *Ae. aegypti* in Harris County, Texas (Houston), and Maricopa County, Arizona (Phoenix), using a nanopore-based third-generation sequencing protocol to resolve host usage and multiplicity. Using this method, approximately 80% of samples from each location with evidence of blood feeding resulted in blood meal identification, with a single host blood meal identified in about 80% of samples and approximately 20% containing evidence of multiple blood meals. Overall, we observed a wide host range with human DNA being the most prevalent followed by feline (*Felis catus*) and canine (*Canis lupus*). We also identified avian, rodent, ungulate and even ectotherm usage by *Ae. aegypti* from Maricopa County. This study demonstrates the utility of a third-generation sequencing method to identify less common hosts, like ectotherms in Arizona, and to resolve multiple feedings which remain difficult to resolve by current Sanger-based methods.

## Simple Summary

The yellow fever mosquito, *Aedes aegypti*, is a vector of several viral pathogens that frequently cause human disease but many of these pathogens are not controlled by vaccination. Effective control of this mosquito is thus critical for reducing disease transmission, and better understanding of the ecology of this species helps to improve the efficacy of operational control. In this study, researchers used a deep sequencing method to examine host usage in two large US metropolitan areas, Houston, TX, and Phoenix, AZ, both with substantial *Ae. aegypti* infestations, using samples collected during standard surveillance activities. In Houston, we identified occasional multiple blood feedings by individual mosquitoes and that the primary host sources were humans and their pets. Results from Phoenix indicated broader host usage, including feeding on livestock, birds, and even reptiles. This study demonstrates the benefits of a deep sequencing method for disambiguating multiple host feedings and detecting the broad scope of host usage.

## 1. Introduction

Both host usage and host range are key factors in cycles of mosquito-borne pathogen transmission [1]. Host usage, or host utilization, is defined as the preference for certain species and the frequency that a certain host is used [2], while host range is the behavioral plasticity to shift from a preferred host to an available one, expanding the number of species, genera, or families utilized in relation to availability in the environment [1,3]. Pathogen transmission cycles are better understood when there is adequate knowledge of the identity and frequency of hosts that are chosen by mosquitoes for blood meals [4,5]. Blood meal analysis (BMA) assists in reaching a comprehensive understanding of what hosts are being utilized and the relationships between hosts and mosquito-transmitted zoonotic pathogens [6].

Various blood meal identification methods have been used, and each has benefits and limitations. For decades, BMA studies were conducted using several antibody-based methods that required specific antibodies for each host or host family being assessed [7,8,9,10,11,12,13]. Antibody-based methods are sensitive but necessarily limited in host identification by the range of host-specific antibodies available; to identify usage of a host, it requires the foreknowledge to include an antibody to detect the host. An increasingly common BMA method involves the selective amplification of host DNA by polymerase chain reaction (PCR) with specific primers, most commonly amplifying the cytochrome c oxidase subunit I (*COI*) gene, with subsequent sequencing of the amplicon using the Sanger method [2,14,15,16,17,18]. These DNA-based methods increase the possibility of identifying hosts by comparison to large sequence databases and have increased the understanding of host usage, often allowing identification to the species level (sometimes with surprising findings). The identification of annelid blood in *Uranotaenia* mosquitoes expanded the known host range and was made possible by this method [2]. The Sanger method is unable to differentiate multiple blood meals taken by a single mosquito without extensive additional efforts (i.e., cloning to isolate individual host sequences). It can also co-amplify mosquito *COI* (which can obscure the result) and can be negatively impacted by the scope and quality of the *COI* databases used for identification [16]. Application of next-generation and third-generation sequencing (TGS) methods have overcome some of the limitations of identification by Sanger-based sequencing. These methods excel in disambiguating multiple host feeding and the impact of co-amplification of mosquito DNA by assigning individual reads from the sample to different hosts [19,20]. While they do not overcome database issues and they still require careful quality control to filter spurious read assignments, deep sequencing methods successfully identify a wide range of hosts [21,22].

*Aedes aegypti* is the primary vector of Zika, dengue, yellow fever, and chikungunya viruses, making it one of the most medically important mosquitoes [12]. It is an important vector of pathogens in many countries around the globe. The adaptivity of *Ae. aegypti* has enabled it to go from a primarily sylvatic species, with access to a wide range of hosts, to a more urban one, where potential hosts are more limited to humans and domesticated animals like pets and livestock [23]. Studies of the feeding behavior of *Ae. aegypti* have demonstrated that it is anthropophilic but has significant plasticity in host usage (reviewed in Olson et al. [24]). Previous studies on *Ae. aegypti* often identify feline (genus: *Felis*) and canine (genus: *Canis*) hosts in addition to human and, less commonly, other mammalian and avian (Class: Aves) hosts [9,17,24,25,26]. Studies of *Ae. aegypti formosus*, considered the original sylvatic form, revealed the wide host range expected of a mosquito that is endemic where humans are infrequent [9,20]. A similarly broad host range has been shown in the more domesticated form of *Ae. aegypti* from northern Mexico, which identified the presence of reptile blood [21].

The variability of host usage and host range found in these previous studies, combined with limited studies in *Ae. aegypti* found in the US, led us to the present study [17,18,24,27]. Our objectives were two-fold: first, to refine a nanopore TGS-based method that could detect blood meals in the presence of substantial mosquito DNA, and then to utilize this method to determine host usage and the frequency of host multiplicity of *Ae. aegypti* from normal vector control surveillance collections rather than collections done specifically for BMA. This study expands our understanding of *Ae. aegypti* host usage in the US and provides researchers with a flexible tool to enhance blood meal identification capabilities in mosquitoes.

## 2. Materials and Methods

### 2.1. Sample Collection

**Figure 1:**
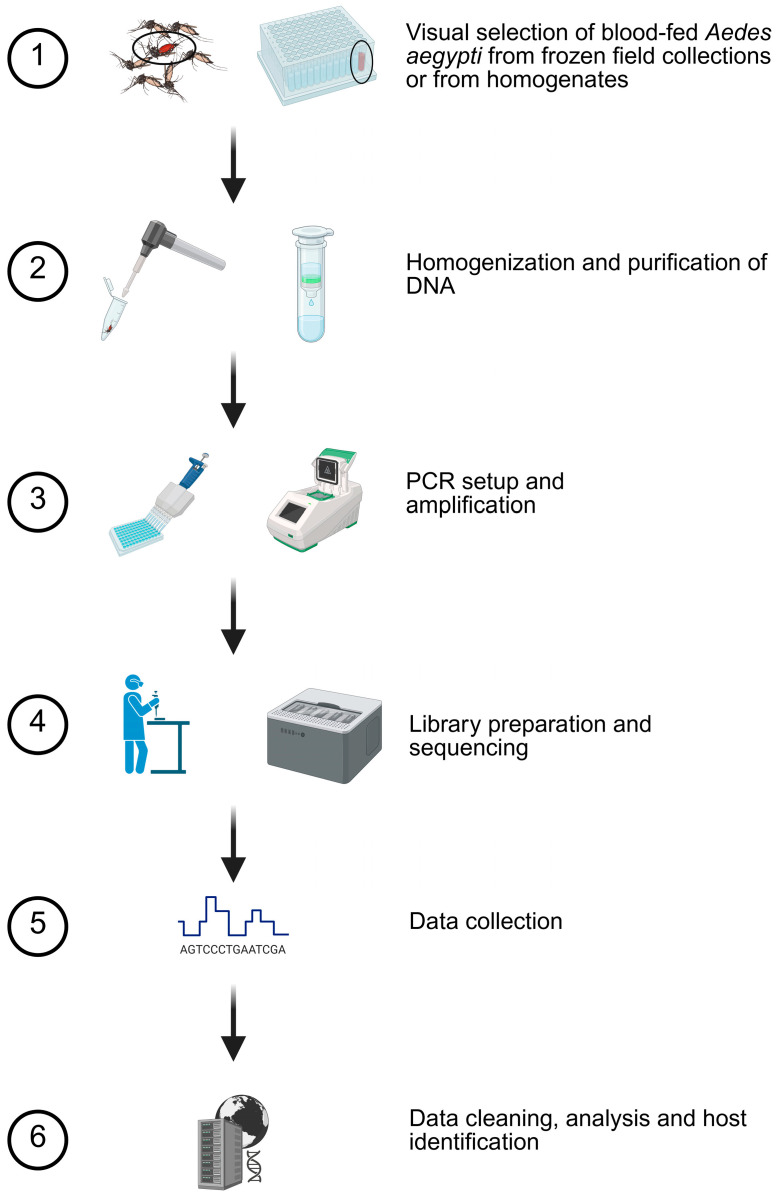
Blood meal analysis methodology. Based on preliminary studies, a six-step procedure was used to determine host usage. Mosquitoes with evidence of blood were selected for homogenization and purification. Specific details are provided in the Methods Section. Created in BioRender. Estep, A. (2026) https://BioRender.com/m4ubsfn (accessed on 28 December 2025).

Mosquitoes were collected in Harris County, TX, using BG-Sentinel traps (Biogents AG, Regensburg, Germany) by Harris County Mosquito and Vector Control Division (MVC Division) employees from June–September of 2019. Traps were sited in densely populated urban/semi-urban locations with a housing stock of single-family homes generally more than 30 years old. Samples were preserved on ice in the field and then identified morphologically to species while under cold anesthetization at the MVC Division. Samples were stored at −80 °C and then shipped frozen to the USDA-ARS-CMAVE where *Ae. aegypti* pools were separated and homogenized as individual mosquitoes for standardized insecticide resistance genotyping [28,29]. Sample homogenates that had an orange or red hue, indicative of the presence of a blood meal, were refrozen at −80 °C for use in this study (Figure 1, Step 1). A total of 31 of the 440 mosquito homogenates appeared positive for blood meal presence. Three mosquitoes with no evidence of a blood meal were extracted to be used as non-blood-fed controls.

Mosquitoes from the metropolitan area of Phoenix, AZ, were collected using encephalitis vector surveillance/carbon dioxide-baited traps (Clarke, St. Charles, IL, USA) or BG-Sentinel traps (Biogents AG, Regensburg, Germany) from May–October 2021 and then identified to species by Maricopa County Environmental Services Department personnel [30,31]. Collection locations were primarily semi-urban/suburban with a range of lots including those large enough to host livestock. Traps were set at ~1:00 PM, allowed to run overnight, and collected at ~10:00 AM the next morning. Frozen *Ae. aegypti* were shipped to USDA-ARS-CMAVE for assessment of genetic insecticide resistance markers. Approximately 5000 *Ae. aegypti* were screened for the visible presence of a blood meal in the abdomen (Figure 1, Step 1) and the 238 that appeared blood-fed were stored at −80 °C until processing.

### 2.2. Preliminary Method Development

Preliminary studies were conducted to determine an optimal amplification temperature, to pilot the use of unpurified PCR products as direct input into the nanopore sequencing library preparation procedure, and to define quality control procedures and thresholds to ensure accurate assignment of reads. Details of these studies are in the Supplementary Information (File S1). During the course of this study, an independently conducted nanopore-based method for host identification was published by Kothera [22] for *Culex* mosquitoes.

### 2.3. Nucleic Acid Purification and Amplification

DNA from the Harris County samples was purified using a silica column kit (gDNA kit, Zymo Research, Irvine, CA, USA) from homogenates (300 μL) diluted in lysis buffer (1200 μL) and processed per the manufacturer’s protocol for liquid samples. Maricopa County *Ae. aegypti* were manually homogenized in 350 μL of lysis buffer using a RNase-free disposable pellet pestle (ThermoFisher, Waltham, MA, USA) or mechanically using 2.0 mm zirconia beads (BioSpec Products, Bartlesville, OK, USA) (Figure 1, Step 2). DNA was purified using the Quick-DNA/RNA Miniprep Kit or the Quick-DNA Miniprep Kit (Zymo Research, Irvine, CA, USA) following the manufacturer’s instructions. DNA was eluted in 50 μL and frozen for downstream PCR and sequencing.

Cytochrome oxidase I PCR was conducted using the primers, amplification conditions, and modified methods from Reeves et al. [16] (Figure 1, Step 3). Amplification was performed in 20 µL reactions using 2X Apex Taq RED Master Mix (Genesee Scientific Corp., San Diego, CA, USA), primers VertCOI_7194_F 5′-CGMATRAAYAAYATRAGCTTCTGAY-3′ (0.47 µM) and Mod_RepCOI_R 5′-TTCDGGRTGNCCRAARAATCA-3′ (0.47 µM), and 1 μL of DNA template at the following conditions: 95 °C for three minutes, followed by 40 cycles of 95 °C for 40 s, 50 °C for 30 s, and 72 °C for three minutes, with a final extension step of 72 °C for seven minutes. Amplicons were visualized on 1% agarose gels in tris–acetate–EDTA buffer using GelRed (Biotium, Inc., Fremont, CA, USA) alongside TrackIt 100 bp DNA Ladder (ThermoFisher, Waltham, MA, USA) and imaged on an iBright system (ThermoFisher, Waltham, MA, USA). The expected amplicon size, including the primers, was 440 bp.

### 2.4. Nanopore Sequencing

Based on successful preliminary nanopore sequencing trials (File S1), unpurified PCR amplicons were used with the R10 chemistry native barcoding kit 96 V14 (LSK-SQK-NBD114.96, protocol version: NBA_9170_v114_revL_15Sep2022). MinKNOW software (versions 23.07.5 and 23.07.12) was used to manage sequencing and base calling on a GridION device (Oxford Nanopore Technologies, Oxford, UK). The manufacturer’s protocol was followed for sequencing sample preparation with one modification: 3 μL of unpurified PCR product was used as input into the end preparation step (rather than 50 femtomoles as directed by the manufacturer’s protocol) (Figure 1, Step 4). Samples were sequenced in batches of 96 samples per flow cell for a maximum 72 h sequencing period and a minimum quality threshold of 10 was set in the MinKNOW software (Figure 1, Step 5).

To allow for a subsequent rigorous quality filtering process that would reduce misassignment of barcodes by MinKNOW and account for the higher error rate of nanopore sequencing (which could lead to misassignment of reads), each individual flow cell contained two controls: one “blood-negative” sample as a barcoded library from a non-blood-fed laboratory *Ae. aegypti* and two “DNA-negative” samples as libraries that only had a barcode but no sample DNA. To further ensure the process was sound, one 96 sequencing run had 8 barcodes purposefully excluded to assess misassignment.

### 2.5. Bioinformatic Analysis

Bioinformatic analysis was conducted on a Dell Xeon laptop (Precision 7520) running Ubuntu 20.04 in Windows System for Linux using the FASTQ and sequencing summary files produced by each nanopore sequencing run. The sequencing summary file was used for initial quality control, and a custom script was used to remove low-quality reads and ensure the presence of barcodes of at least 37 bases on each end of a read and a minimum 95% identity to the known barcode sequence. Following this initial quality control filtering, host identification was conducted by a Basic Local Alignment Search Tool (BLAST version 2.6.0) [32] query against a custom database created from the Barcode of Life Database (BOLD) Systems [33,34] that contained approximately 500,000 chordate *COI* sequences with species names. The resulting output files were filtered again to only include reads with a minimum 97.5% match (as the least strict threshold that resulted in minimal assignments to the two types of control samples) and with an alignment length of at least 400 bases. This threshold was suitable for the error rate of nanopore data at the time of sequencing and slightly above the 97% identity standard for determining genus [35]. This filtered file was then separated by barcode and blood meal identification. The minimum read threshold to be considered a species-level match was two full-length reads (>400 bp) that met the above filters. The sequencing summary files, the BLAST-formatted database, and the pipeline used in this analysis are available in the data repository. Raw sequencing data were submitted under BioProject PRJNA1242810 and SRA accession numbers SAMN47605645-SAMN47605929. Assignments, in some cases to the species level, for each sample are in File S2. Verification of the mosquito species is possible by the same method with a database containing mosquito *COI* sequences.

### 2.6. Statistical Analysis

The independence of single-host frequency vs. multiple-host frequency, as well as the independence of feline, canine, and human host frequency between Harris and Maricopa counties, was analyzed using Fisher’s Exact test in R version 4.4.2 [36].

## 3. Results

### 3.1. Nanopore Sequencing and Quality Control

Sequencing results indicated that the use of unpurified PCR amplicons resulted in successful output of data but as expected, output was lower than when using purified amplicons. Rather than the manufacturer’s expected output of 10–20 Gbases per flow cell, we averaged 7.13 ± 3.20 Gbases and 12.15 ± 5.10 M reads per cell across three sequencing runs (File S1). Estimated N_50_ was consistent at 564.7 ± 1.2 bases. Approximately 84.6% of reads met the initial quality filtering by the MinKNOW software. Barcode separation was also managed by the software and 88.3 ± 2.5% of reads were assigned to barcodes. Individual sequencing run reports are provided in the data repository.

Although the MinKNOW software conducted an initial quality filter and binning by barcode, we performed additional quality control filtering to ensure that we could screen out misbinning (either due to barcode crosstalk or the higher native error rate) and thus reduce erroneous findings. For eight barcode blanks (barcodes not included in the library preparation), an average of 970 ± 151 reads were assigned to barcodes by the software. Examination of the sequencing summary file for these reads showed most assignments were based on short barcode matches, often on one end and of relatively low identity. The secondary filtering conducted as described in the Methods Section (requiring a barcode match of at least 37 bases and higher identity) removed over 90% of misassigned reads to result in an average of 93 ± 28 remaining reads. Among those passing reads remaining after secondary filtering, few remaining reads resulted in assignment (average = 0.375 reads/library). This represents misassignment of less than 0.038% (0.375 assigned reads/970 prefiltered reads) of the average number of reads per barcode, which indicates that the quality control steps removed nearly all the spurious assignments (File S1). With respect to the duplicate NFW-only control libraries included in each sequencing run, only two assigned reads passed the filters. When this same filtering process was applied to all the barcoded libraries present in the study, an average of 39.66 ± 8.29% of reads were retained. Notably, substantial amplification of the *COI* sequence of the mosquito occurred (File S1) using the *COI* primers, which provide ample data to verify the species of the mosquito if uncertainty exists. We also observed that reads mapping to the blood meal versus the mosquito averaged 5.7% of the filtered reads (median 0.8%) with wide variation from 0 to 56.2% (File S2).

### 3.2. Ae. aegypti Host Multiplicity

**Figure 2:**
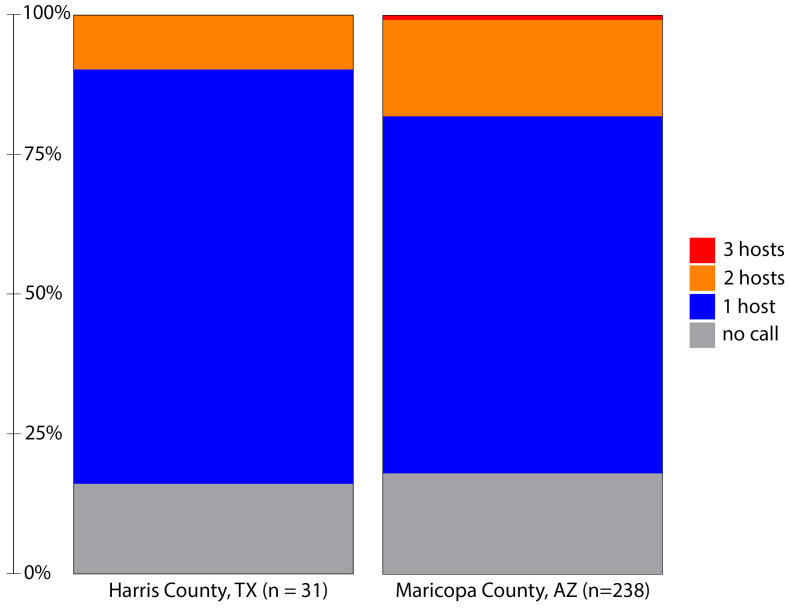
Host identification success and host multiplicity in blood-fed *Aedes aegypti* collected from Harris County, Texas, in 2019 and Maricopa County, Arizona, in 2021 using a nanopore-based sequencing method. The minimum threshold for host identification is defined as at least two sequences with alignment length > 400 bases and identity to the host cytochrome oxidase I gene of greater than 97.5%. Initial filtering required barcodes at each end >37 bases in length and >95% identity. Note that three hosts were identified in two samples from Maricopa County, Arizona.

One purpose of this study was to utilize a TGS method for BMA, allowing us to easily disambiguate multiple blood meals. While Sanger sequencing and antibody-based methods can detect multiple feedings by clone sequencing and host-specific antibodies (respectively), deep sequencing-based methods have shown promise to easily detect multiple blood meals as host identification is made on individual reads and compared against the combined sample breadth provided by large databases like NCBI and BOLD [20,21]. In this study, we identified hosts from 81% (25/31) of the blood-fed samples from Harris County, TX (Figure 2). Of the 25 samples with a blood meal identification, a single host was identified for 22 samples, and two hosts were indicated for three samples.

Initial visual screening of Maricopa County samples identified 238 *Ae. aegypti* with evidence of a blood meal. Using this method, we determined a blood meal for 82% (195/238) of these samples (Figure 2). As with the samples from Texas, most samples indicated feeding on a single host; however, 43 samples contained DNA from multiple hosts. In two cases, we identified the presence of DNA from three distinct hosts: one with feeding on human, feline and avian hosts and another with evidence of feeding on human, canine and livestock hosts. Multiple host feeding has been observed in populations of *Ae. aegypti* from several locations [20,24]. A comparison of Harris and Maricopa County host multiplicity was not statistically different (Fisher’s Exact test; *p* = 0.1791).

### 3.3. Host Usage in Ae. aegypti from Harris County, TX

**Figure 3:**
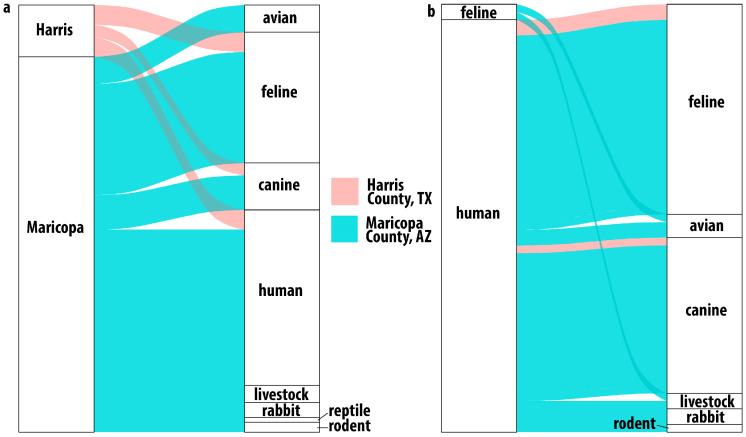
Host usage in Harris County, Texas, and Maricopa County, Arizona. Alluvial plot showing (**a**) single host identifications. All samples from Harris County identified feline, human or canine hosts while Maricopa County results indicated a broader host range including birds, livestock and even reptiles. Multiple host usage (**b**) was observed in both locations. In Harris County, the combinations were human with feline or canine while in Maricopa County, the combinations were much broader.

Among the Harris County mosquitoes with evidence of having taken a blood meal, sequence data showed that these *Ae. aegypti* were frequently feeding on felines (44%), humans (28%), and canines (20%) (Figure 3a). Three mosquito blood meals contained DNA from multiple hosts: two indicated feeding on human and feline, and one indicated human and canine (Figure 3b). In these samples, reads assigned to human DNA were more abundant than the reads assigned to feline or canine. Previous BMA studies have shown a similar limited host range [12,18].

### 3.4. Host Usage in Ae. aegypti from Maricopa County, AZ

Detected host usage in Maricopa County was broader than that observed in Harris County (Figure 3a,b) and shows, as with Harris County, that these *Ae. aegypti* are not as strongly anthropophilic as might be expected based on previous studies [4,5,11,12,21,37,38]. While human blood (113/195), feline blood (72/195), and canine blood (33/195) represented most of the host identifications in Maricopa County, the next most frequent host group was birds (15/195), with numerous detections of feeding on chickens (10) (*Gallus gallus*) and several identifications of feeding on owls, including the great horned owl (4), *Bubo virginianus*, and the long-eared owl (1), *Asio otus*. We also identified feeding on members of the *Zenaida* genus of doves (3). We identified one detection of feeding on a hawk of the *Accipiter* genus, of which *Accipiter atricapillus* is present in the Phoenix area [39].

We detected feeding on rodents (5), several common livestock animals (8), and rabbits (10), which have been identified as hosts in other studies of *Ae. aegypti* feeding. We also detected feeding on ectotherms, with one identification of the desert tortoise, *Gopherus agassizii,* and one identification of feeding on the ornate tree lizard, *Urosaurus ornatus*.

The results from Maricopa County also indicate that taking multiple blood meals was common in these *Ae. aegypti*. Twenty-eight of the 40 multiple-host samples had human and feline or canine blood, likely from being domiciled together and thus representing a convenient source of blood. Some of the other dual-host results were more surprising; one sample had both feline and bird DNA present, and another had both feline and livestock DNA present. Notably, we detected two samples with evidence of three host feedings.

## 4. Discussion

**Figure 4:**
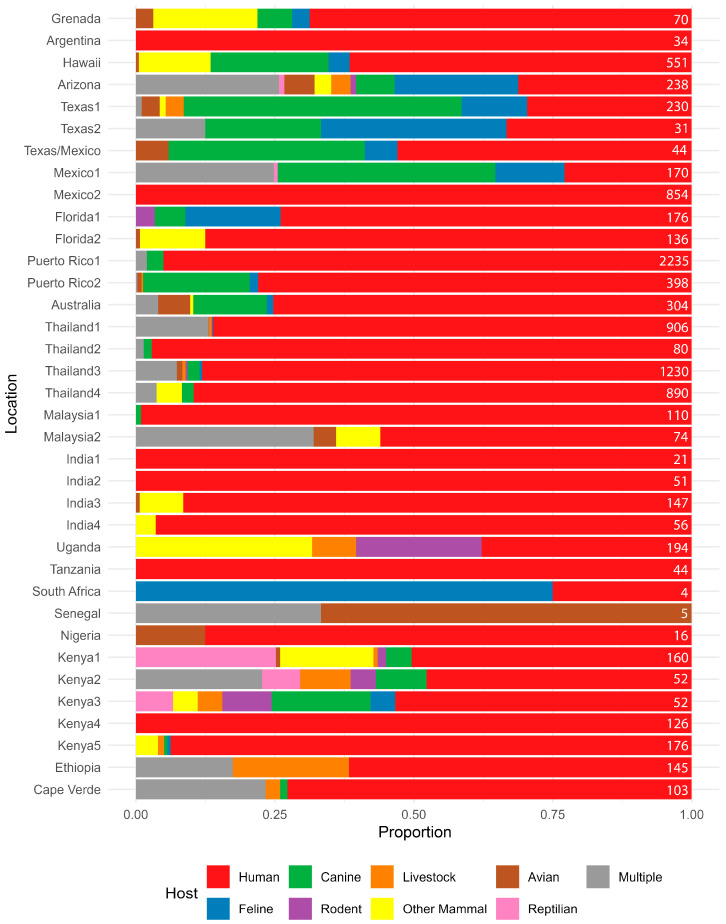
Summary of host usage studies on *Aedes aegypti*. Values are expressed as a percentage, and the geographic location of the study is listed on the left. Results from this study from Harris County, TX, and Maricopa County, AZ, are denoted as Texas2 and Arizona, respectively. Data sources are: Grenada [43], Argentina [44], Hawaii [11], Texas1 [24], Texas/Mexico [27], Mexico1 [21], Mexico2 [45], Florida1 [18], Florida2 [17], Puerto Rico1 [37], Puerto Rico2 [14], Australia [25], Thailand1 [12], Thailand2 [46], Thailand3 [5], Thailand4 [26], Malaysia1 [10], Malaysia2 [47], India1 [10], India2 [10], India3 [48], India4 [49], Uganda [9], Tanzania [50], South Africa [51], Senegal [15], Nigeria [7], Kenya1 [9], Kenya2 [20], Kenya3 [20], Kenya4 [8], Kenya5 [52], Ethiopia [53], and Cape Verde [13]. The number at the right of each line represents the number of samples used to calculate the percentages.

Across both counties examined in the study, human DNA was found in approximately 50% of all the *Ae. aegypti* blood meals identified, followed in decreasing amounts by feline and canine. Single-host blood meals for feline, canine and human hosts did not differ between the sites (Fisher’s Exact test; *p* = 0.4216). We did identify a broader host range in the samples from Maricopa County where we detected feeding on a variety of other mammals and even reptiles, but we are hesitant to ascribe any real significance when we consider that sample numbers varied between areas and also in the context of other BMA studies (Figure 4) in *Ae. aegypti,* which found that both host multiplicity and host range can vary widely. Some reports show humans are hosts for *Ae. aegypti* exclusively, while others show little to no feeding on humans (Figure 4). Similarly, some studies indicate one host, a few hosts, or a wide variety of hosts (Figure 4). Three and even four host feedings have been previously reported in field-collected *Ae. aegypti* and in laboratory studies [12,40].

As noted previously, *Aedes aegypti* have demonstrated high adaptability by movement from a native sylvatic environment to an invasive urban environment [41,42]. Thus, these variations in hosts could be due to a variety of reasons, including limitations of the method (limited anti-host antibodies and inability to parse multiple overlapping Sanger electropherograms), limitations in host usage due to collection site selection (inside houses versus green space collections) or limitations in analysis (missing species-specific sequences in databases). The differences we observed between Houston and Phoenix may be the result of differences in host availability and convenience rather than actual differences due to preference, but we were not able to assess these factors in this study.

The detection of a diverse range of hosts in Maricopa County may also provide insight into ecological strategies implemented by *Ae. aegypti* to survive inhospitable environments, though we could not rigorously assess this within the confines of this study. We note that recent studies from Northern Mexico, with a presumably similar climate to Arizona, showed bird and reptile host usage [21,27]. Evidence of feeding on livestock, birds, rodents, and even reptiles may indicate *Ae. aegypti* could be sheltering in cryptic outdoor locations like livestock barns, chicken coops, burrows, and in vegetation away from direct exposure to daytime temperatures above 40 °C and from exposure to low humidity. Notably, six samples were positive for equine blood, and these were collected from three distinct sites. Visual examination of satellite imagery indicates evidence of horses within 150 m of the trap location for two of the three sites.

Considering the *Ae. aegypti* host range studies (Figure 4), there is evidence that multiple host feeding is relatively common in *Ae. aegypti* and was likely underreported as antibody-based methods, the primary methods employed for many years, and Sanger-based methods, the current commonly used methods, are both limited in their ability to detect multiple feedings without additional testing. While NGS and TGS methods can improve multiple feeding detection, they cannot disambiguate multiple feedings on the same species, which may be common in *Ae. aegypti,* using *COI* alone [40,54]. We also note that this method could be easily modified to include assessment of other genes of public health importance, like pathogens or genetic resistance factors.

Next-generation sequencing and TGS-based methods of BMA have several advantages over traditional methods and are increasingly common [19,20,22,55,56]. Like Sanger-based BMA, the ability to identify unexpected hosts improves as the number of species-specific *COI* sequences available in public databases continues to increase. In this study we used only about 500,000 well curated chordate sequences from the approximately 9 million species-specific *COI* sequences available in the latest version of the BOLD.

Mass sequencing methods for BMA do not require specific sample types, such as excised blood-filled abdomens [16]. The methods developed for this study used DNA isolated from whole individuals, such as is commonly available to mosquito control programs. This provides more utility for samples used during routine pathogen testing and insecticide target-site resistance studies, which are commonly the primary efforts of *Ae. aegypti* surveillance. Often, the presence of a blood meal is not detected until samples have been homogenized, at which point the host and mosquito DNA have mixed and the host DNA is highly diluted, and host amplicons are rare relative to the quantity of mosquito amplicons after PCR. The TGS method described in this study is very sensitive to low host amplicon sequences among many mosquito sequences as we could detect host reads at less than 1:10,000 (File S2).

The use of TGS-based BMA also allows for the resolution of multiple hosts, which is not easily possible using the traditional Sanger-based method as multiple host DNA signals result in multiple ambiguous peaks in the electropherograms. Unintended amplification of mosquito *COI* by putatively vertebrate-specific primers as we saw in our study (see Supplementary Files) would result in ambiguous Sanger results but can be screened out bioinformatically since host and mosquito sequence data are easily parsed. While amplification of the mosquito DNA is detrimental for Sanger-based BMA, using a whole mosquito homogenate for deep sequencing is of benefit as it allows for molecular identification of the mosquito species, thereby decreasing the need for morphological identification and uncertain speciation from damaged or dubiously identified samples.

The use of mass sequencing data for BMA does require consideration and mitigation of the limitations that are common to any database comparison method. The ability to make identifications is limited by the expansiveness of the database used for comparison. Outdated or poorly curated reference databases and preconceived assumptions of blood-feeding behavior (such as that mosquitoes only take blood from vertebrates and thereby excluding taxa such as annelids) will exclude potential BLAST matches [2]. Also, careful determination of thresholds for quality filtering needs to come into play to determine whether an identification is accurate to the genus or species level. The detection of a hawk as a host in Maricopa County is a good example: our database identified the reads as from *Accipiter (Astur) gundlachi*, a hawk endemic to Cuba. Closer examination of the data shows two limitations of the database comparison method. First, *Accipiter atricapillus*, which is present in Arizona, is not in the database [39]. Second, as a matching method, the sequences for *Accipiter (Astur) gundlachi*, which were in the database, were the best match possible but at levels just above the threshold. This result illustrates that discretion must be used when determining species- and genus-level identifications and it must be acknowledged that species are missing from databases.

This study demonstrates that implementing a TGS-based method is relatively easy and can improve some of the challenges of Sanger-based methods but that the data obtained must be carefully screened for erroneous sequences using several layers of quality control filtering and the development of analysis pipelines that can remove misassignments. We expect that as nanopore sequencing accuracy continues to increase, the ability to assign accurately will improve.

## Data Availability

All data supporting this manuscript are included in this publication and the Supporting Files. Sequences underlying this study have been submitted to NCBI under Accession Number: PRJNA1242810.

## Supplementary Materials

The following supporting information can be downloaded at: https://www.mdpi.com/article/10.3390/insects17020175/s1. File S1. Supplemental and preliminary methods used to develop the protocols included in this study and to verify the effective use of unpurified PCR products at the initial step of sequencing library preparation. File S2. Data including sample metadata, sequencing data, and the counts attributed to each species-level identification.

## Abbreviations

The following abbreviations are used in this manuscript:

BMA	Blood meal analysis
PCR	Polymerase chain reaction
*COI*	Cytochrome oxidase I
TGS	Third-generation sequencing
MVC	Mosquito and Vector Control Division
USDA-ARS-CMAVE	United States Department of Agriculture, Agricultural Research Service, Center for Medical Agricultural and Veterinary Entomology
