# Supplementary material for: Host Usage in Aedes aegypti from Houston, Texas, and Phoenix, Arizona, Using Third-Generation Sequencing Blood Meal Analysis
Source: Insects. 2026 Feb 5;17(2):175. doi: 10.3390/insects17020175 (PMC12940420; doi:10.3390/insects17020175)
Supplement: Supplementary file 1 [file insects-17-00175-s001.zip › S1 File.pdf]

S1 File. Supplemental and preliminary methods used to develop the protocols included in this study and to verify the effective use of unpurified PCR product at the initial step of sequencing library preparation

Blood meal detection time series

Five to ten day old females from two laboratory strains of *Ae. aegypti* (Orlando and Puerto Rico) were used for this study. Six non-blood fed mosquitoes were aspirated from the cage before blood exposure and used as negative controls. Mosquitoes were given access to warmed, excess human blood in a screened cages. Groups of six mosquitoes were aspirated from the cage while actively feeding, fully engorged, and then 24 or 48 hours after blood feeding. All mosquitoes were frozen at -80 ºC until processing. Three randomly selected mosquitoes from each of the five conditions were individually homogenized in 350 µL of lysis buffer and DNA was extracted using the Zymo Research Quick-DNA Miniprep Kit (Zymo Research, Irvine, CA). DNA was eluted in 50 µL of nuclease free water (NFW) and stored at -80 ºC. Amplification was performed in 20 µL reactions using 2.0X Apex Taq RED Master Mix (Genesee Scientific Corp., San Diego, CA), primers VertCOI_7194_F 5'- CGMATRAAYAAYATRAGCTTCTGAY- 3' (0.47 µM) and Mod_RepCOI_R 5'- TTCDGGRTGNCCRAARAATCA -3' (0.47 µM)[7], and 1 uL of DNA template (Reeves et al., 2018b) at the following conditions: 95 °C for three minutes, followed by 40 cycles of 95 °C for 40 seconds, 45 °C for 30 seconds, and 72 °C for three minutes, with a final extension step of 72 °C for seven minutes. Amplicons were visualized on 1% agarose gels in tris-acetate EDTA buffer using GelRed (Biotium, Inc., Fremont, CA)

alongside TrackIt 100 bp DNA Ladder (ThermoFisher, Waltham, MA) and imaged on an iBright (ThermoFisher, Waltham, MA).

Host amplification optimization

Additional PCR was conducted using the same templates and methods described above at annealing temperatures of 45 °C, 50 °C, 53 °C, and 54 °C to determine optimal amplification conditions. Five microliters of each amplicon was electrophoresed as described above.

PCR reactions with annealing temperatures of 45°C and 50°C were cleaned using the Quick-DNA Miniprep Kit and sequenced using the Oxford Nanopore SQK-LSK109 sequencing kit (protocol version NBA_9093_v109_revF_12Nov2019) with native barcoding expansion kits (EXP-NBD104 and EXP-NBD114) (Oxford Nanopore Technologies, Oxford, England). A working protocol is in in the data repository at DOI: 10.15482/USDA.ADC/26018947.

Supplemental results

Use of unpurified PCR amplicons as input for sequencing

Purified DNA from field collected, blood fed Harris County, Texas *Ae. aegypti* was amplified as above at an annealing temperature of 50 °C. To test whether additional purification was necessary before sequencing, 16 samples were then directly used, without any PCR cleanup, as input into the Nanopore sequencing protocol described above. Samples were barcoded and sequenced following the manufacturer instructions. Reads were binned by the MinKNOW software and then used to determine

While sorting the Maricopa samples for the presence of blood meal, it was observed that few were fully engorged and that many appeared to contain well digested meals. In the study by Reeves et al. (2018b), well digested samples gave generally low amplification success and thus it

was required that we determine the length of bloodmeal digestion time that would still result in a successful detection of blood meal source. To examine this, we processed laboratory *Ae. aegypti* at differing time intervals of blood meal digestion (24 and 48 hours). We sequenced COI amplicons from three mosquitoes from each of five conditions; non-blood fed, partially blood-fed (where blood feeding was interrupted before engorgement), fully engorged, 24 hours post-blood feeding, and 48 hours post-blood feeding. This experiment also allowed us to examine whether functional sequencing quality (enhanced assignment of reads) increased during the length of the sequencing run or if we could use a considerably shorter sequencing period.

First, statistical comparison to a database containing mosquito and human COI showed that reads collected in the initial portion of the sequencing run (reads 4000-7999) were not assigned differently than those collected later in the run (reads 76,000-79,999) based upon Pearsons's chi-square test of independence. Non-blood fed mosquitoes in the first file of reads vs. the twentieth file of reads ($\chi 2 = 3.098$, df = 2, P = 0.2125), partially blood fed mosquitoes in the first file of reads vs. the twentieth file of reads ($\chi 2 = 1.9401$, df = 2, P = 0.3791), fully engorged mosquitoes in the first file of reads vs. the twentieth file of reads ($\chi 2 = 0.079692$, df = 2, P = 0.9609), 24 hour post blood fed mosquitoes in the first file of reads vs. the twentieth file of reads ($\chi 2 = 1.1653$, df = 2, P = 0.5584), and 48 hour post blood fed mosquitoes in the first file of reads vs. the twentieth file of reads ($\chi 2 = 0.14724$, df = 2, P = 0.929). This was true for each of the five conditions we tested and for both the mosquito and human read assignment. Second, we observed extensive assignment of reads as human from the interrupted feeding, fully engorged, and 24 hours post-blood meal groups; there was little recovered blood meal DNA from 48 hours post-blood meal and the reads that were detected were at the same background level as the no blood meal group. Third, by comparing the same samples at two different amplification

temperatures we observed that more reads were assigned to the host DNA versus the mosquito DNA at an annealing temperature of 50 °C rather than 45 °C. Thus, the annealing temperature of 50 °C is the optimal amplification condition for our method. Fourth, we observed that a small number (less than 0.2%) of reads were attributed to human even in samples that had not taken a blood meal. These human reads could represent contamination from the process but likely represent misassignment of reads into barcodes since the MinKnow software uses a barcode binning threshold of around 80% without requiring a full-length barcode. This indicated that we needed to conduct additional quality control processing of the reads to reduce misassignment before comparison to the database for species assignment (Li et al., 2019).

In this preliminary study, we also observed that the database for host assignment needed to be broad and contain multiple sequences for each species to represent the variation in COI sequences possible within a species, which has been estimated at 0.6% in humans and up to 1.8% in other species. Our initial human COI sequence (HG800481.1) identified very few sequences in the data but the inclusion of a sequence from human mitochondrial haplotype P (MN849799.1) detected 100s to 1000s more reads from the same data. This was an important observation that led us to create a database that included all available COI genes of chordates to incorporate as much sequence diversity as available to not miss any host blood meals present.

Optimization of amplification conditions

When assessing the results of the blood digestion time series we observed that the no blood meal samples produced bands of the expected size with primers designed to amplify vertebrates and many of these were assigned to the mosquito. This indicates that the primers are not vertebrate specific and readily amplify mosquito COI. While the methods of Reeves et al. (2018b) excludes the majority of mosquito DNA by only using blood released from mosquito

abdomens for DNA extractions, whole mosquito samples (like those commonly available at mosquito control programs), contain an abundance of mosquito DNA relative to the blood meal source. To minimize the amplification of mosquito DNA and maximize the amplification of host DNA we tested multiple annealing temperatures in addition to that used by Reeves (2018b). Although an annealing temperature of 50°C versus 45°C resulted in less spurious amplification, annealing temperatures above 50°C resulted in reduced overall amplification. Therefore, annealing at 50 °C was used for subsequent experiments.

Use of unpurified PCR products for nanopore sequencing

The standard nanopore sequencing protocol specifies purified amplicon DNA as the source material but purifying hundreds of samples is time consuming and costly. We examined whether it was necessary clean up the PCR reaction after amplification and before input into the sequencing and barcoding procedure. To assess this, we amplified COI from Harris County blood fed *Ae. aegypti* mosquitoes previously genotyped for insecticide resistance markers (Estep et al., 2023). These amplicons were used as input into the Nanopore sequencing procedure without post-PCR purification. All samples tested produced sufficient numbers of sequencing reads with the lowest output near 4,000 total reads. Amplicons were taken directly into the Nanopore barcoding procedure without the need for cleanup in subsequent sequencing runs.
